# Measurement Invariance of Perfectionism Measures in Students with and without a History of Non-Suicidal Self-Injury

**DOI:** 10.3390/ijerph181910171

**Published:** 2021-09-28

**Authors:** Kate E. Tonta, Mark Boyes, Joel Howell, Peter McEvoy, Penelope Hasking

**Affiliations:** 1 Faculty of Health Sciences, School of Population Health, Curtin University, Perth 6102, Australia; Kate.Tonta@curtin.edu.au (K.E.T.); Mark.Boyes@curtin.edu.au (M.B.); Joel.Howell@curtin.edu.au (J.H.); Peter.McEvoy@curtin.edu.au (P.M.); 2 Curtin Enable Institute, Faculty of Health Sciences, Curtin University, Perth 6102, Australia; 3 Centre for Clinical Interventions, Perth 6003, Australia

**Keywords:** perfectionism, measurement invariance, non-suicidal self-injury

## Abstract

Perfectionism is a transdiagnostic process which may be implicated in the onset and maintenance of non-suicidal self-injury. No study has evaluated whether reported differences in perfectionism between individuals with and without a history of self-injury represent genuine group differences or measurement artefacts. The present study reports an investigation of the measurement invariance of two common scales of perfectionism, the Frost Multidimensional Perfectionism Scale-Brief (FMPS-Brief) and the Clinical Perfectionism Questionnaire (CPQ), among university students (*M*_age_ = 20.48, *SD*_age_ = 2.22, 75.3% female, 22.8% male) with and without a history of self-injury (total *n* = 711). Results revealed full residual error invariance for the two-factor model of FMPS-Brief, while the bifactor model of the FMPS-Brief and the two-factor model of the CPQ demonstrated partial metric invariance. Accounting for partial metric invariance, the bifactor model of the FMPS-Brief also demonstrated partial residual error invariance. The current findings suggest that observed differences using the FMPS-Brief reflect genuine differences in perfectionism between individuals with and without a history of self-injury. Further, while researchers using the bi-factor model can have confidence that the general factor can adequately assess group differences, differential item functioning should be considered if using the strivings and concerns factors. Finally, in the current data, the CPQ did not perform as expected in baseline model fit and future research should replicate assessments of measurement invariance in this measure.

## 1. Introduction

In 2019, more than one in every 100 deaths worldwide was by suicide [1], with a representative sample indicating that over half of the Australian population reported exposure to suicide of someone known to them [2]. The impact of death by suicide includes significant complicated grief and increased risk of depression, anxiety, and suicidal ideation for those exposed to the death. Consequently, suicide prevention is an important target for public health policy and intervention. Understanding risk factors associated with future suicidal ideation and behaviour is critical for prevention and intervention efforts. One such factor is non-suicidal self-injury (NSSI), which is deliberately damaging one’s body tissue without suicidal intent and in a way that is not culturally sanctioned [3]. Although NSSI and suicide are functionally distinct behaviours in that NSSI is specifically *non*-suicidal in intent, frequency of NSSI and the number of methods used to self-injure have been identified in meta-analytic research as important predictors of suicide behaviour [4,5]. The most commonly reported function of NSSI is to regulate intense or unwanted emotions, and NSSI has been identified as an important area for further research [6]. NSSI may present as a range of behaviours including, but not limited to, cutting, hitting, and severe scratching [3]. Although only a small proportion of individuals seek urgent care relating to their self-injury [7,8,9], an estimated 13.4% of young adults in community populations have engaged in NSSI at least once in their lifetime, and this figure is higher (20%) for young adults attending university [10]. These estimates are considered to be a robust representation across a range of cultural contexts, with data from over 230,000 participants in Asia, Australia, New Zealand, Canda, Europe, the UK and the US. In understanding self-injury and suicide, it is important to consider the influence of personality, cognitions, and emotion variables. Personality factors such as higher neuroticism and lower conscientiousness have been associated with suicidal ideation and NSSI [11,12,13]. One important cognitive process that is associated with both suicide [14,15] and engagement in NSSI [16] is perfectionism. Perfectionism, the pursuit of personally demanding high standards in the face of adverse consequences, is a transdiagnostic process associated with a range of psychopathologies, including but not limited to anxiety, mood, eating, and obsessive compulsive disorders [17,18].

Perfectionism has been associated with NSSI in non-clinical samples of adolescents as well as adolescents with clinical diagnoses of eating disorders [19]. In non-clinical adult samples, research using multidimensional models of perfectionism has shown mixed associations, with some subscales of perfectionism (e.g., concern over mistakes) strongly associated, while other subscales showed no significant association (e.g., personal standards) [20]. Although these findings indicate that perfectionism is likely an important process related to NSSI, the measurement of perfectionism is inconsistent across studies, with some research using multidimensional models [20] and others using subscales of larger measures of eating disorder pathologies [19]. Indeed, historically perfectionism has been defined in a number of ways, yet most theories and definitions can be distilled to two core elements: perfectionistic strivings (the setting of personally demanding high standards) and evaluative concerns (self-critical evaluations of self-worth in the context of high standards). Two common measures of perfectionism which align with this conceptualisation are the Frost Multidimensional Perfectionism Scale-Brief [21] and the Clinical Perfectionism Questionnaire [22]. Although both measures are well-validated in the existing literature as two-dimensional [23,24] the argument that a general factor of perfectionism is more parsimonious persists [25]. Bifactor modelling is a statistical technique which may provide information to test this conceptualisation. A confirmatory bifactor model allows examination of both common (i.e., the general factor of perfectionism) and distinct factors (i.e., perfectionistic strivings and perfectionistic concerns) in variance amongst items. Research into both the FMPS Brief and the CPQ has shown that a bifactor model has superior fit compared to single or two-factor models, and that the general factor accounts for much of the shared variance in the bifactor models [25,26].

In addition to the inconsistencies in how perfectionism has been measured in the context of NSSI, it is also important to consider if the nature of cognitive and emotional difficulties associated with NSSI might mean that individuals with lived experience of NSSI respond differently to measures of perfectionism across different domains (i.e., strivings, concerns, and general perfectionism). Specifically, it is important to evaluate how the psychometric properties of perfectionism measures may (or may not) vary between individuals with and without a history of NSSI. Although there is existing research assessing NSSI-related differences in perfectionism [16], to date, none of these studies have assessed measurement invariance. Prior to making meaningful group comparisons, measures should be evaluated within the population of interest to ensure that groups interpret and respond to items in a similar way [27]. If there are systematic differences in the way two groups are interpreting and responding to items, differences in group means are uninterpretable. The use of perfectionism measures that are not psychometrically invariant may lead to false conclusions about processes associated with NSSI, as has been reported with other key processes including emotion regulation, distress tolerance, and rumination [28,29,30].

The present study sought to explore the measurement invariance of two common measures of perfectionism across individuals with and without a history of NSSI. The CPQ and FMPS were subjected to a stepwise bottom-up evaluation of measurement invariance, testing configural (i.e., equal factor structure), metric (i.e., equal factor loadings), scalar (i.e., equal item intercepts), and residual error (i.e., equal residual errors) invariance.

## 2. Materials and Methods

### 2.1. Participants and Procedure

Data for this study were collected as a part of a larger study on emotional health. This study was approved by the [blinded for review] Human Research Ethics Committee. In total, 712 university students participated (*N_CPQ_* = 711, *N_FMPS_* = 708) in this online survey. Recruitment occurred through social media advertising, emails to student organisations at universities, and from a research participation pool. Participants were provided an online information sheet and asked to provide consent by marking a checkbox before proceeding to the survey. Participants were aged between 17 and 34 years of age (*M* = 20.48, *SD* = 2.22); 22.8% were male, 75.3% female, 1.7% identified as another gender, and 0.1% preferred not to disclose. Participants were required to be enrolled at an Australian university. Participants were either included in a prize pool which included an iPad and gift cards to the value of AUD$50, or received course credit in exchange for their participation. See Table 1 for sample characteristics across the two measures.

### 2.2. Measures

#### 2.2.1. Inventory of Statements about Self-Injury

The Inventory of Statements About Self-Injury [31] measures history of self-injury (defined to participants as intentionally harming oneself without intention to suicide). Participants who indicated they had a history of NSSI (i.e., “Have you ever engaged in non-suicidal self-injury?”) were presented with 12 common methods of NSSI (e.g., cutting, burning) and provided a lifetime frequency of each behaviour. NSSI was operationalised as a binary variable (history of NSSI vs. no history of NSSI), and the subsequent 12 items are used for descriptive purposes. The ISAS has been widely used in research and has established test–retest reliability (4-week, *r* = 0.85; 1-year, *r* = 0.68 [32]).

#### 2.2.2. Frost Multidimensional Perfectionism Scale-Brief

The FMPS-Brief [21] is an 8-item measure of perfectionism. This measure has two subscales (perfectionistic striving, e.g., “I have extremely high goals”; and evaluative concerns, “If I fail at work/school, I am a failure as a person”). However, emerging research suggests that a bi-factor model has superior fit to the single or two-factor models, and that the general factor accounts for the majority of the shared variance in the bifactor model [25]. The general factor had strong internal consistency (*α* = 087, ω = 0.87) in this sample, as did the subscales of strivings (*α* = 0.84, ω = 0.85) and concerns (*α* = 0.91, ω = 0.91).

#### 2.2.3. Clinical Perfectionism Questionnaire

The CPQ consists of 12 items assessing thoughts and behaviours relating to perfectionism over the past month. An example item is “Over the past month, have you pushed yourself really hard to meet your goals?”. The factor structure of this measure has been inconsistent. Although initially conceptualised as a unidimensional measure, the CPQ has been considered as two-dimensional according to a range of factor analytic findings [33,34]. These two factors are perfectionistic strivings (e.g., “Have you been told that your standards are too high?) and perfectionistic concerns (e.g., “Have you felt a failure as a person because you have not succeeded in meeting your goals?”). However, there is also evidence to support a bi-factor structure following the removal of the two negatively worded items which appear problematic in their pattern of cross loading [26]. Across all factor structures tested in this study, the negatively worded items were excluded from analyses, consistent with previous findings [25]. Not surprisingly, the strongest model fit statistics emerge when using a bifactor model [25,26], and given the theoretical framework of clinical perfectionism as unidimensional [35], there appears to be sound theoretical basis for this measurement model.

In the present data set, the strivings subscale had acceptable internal consistency (*α* = 0.78, ω = 0.78), whereas the concerns subscale was marginal and lower than expected according to the existing literature (*α* = 0.66, ω =.70). The total scale score also had strong internal consistency (*α* = 0.81, ω = 0.81).

### 2.3. Data Analysis

Analyses were conducted in MPlus Version 8 [36] using maximum likelihood estimation with robust standard errors (MLR). MLR is robust to non-normality and handles missing data using full information maximum likelihood [37]. Model fit was assessed according to the Standardized Root Mean Square Residual (SRMR; close to 0.08 or below), Root Mean Square Error of Approximation (RMSEA; close to 0.08 or below), and a Comparative Fit Index (CFI; in the 0.90–0.95 range or higher) [38]. 

Measurement invariance was assessed through multiple groups confirmatory factor analysis (estimated with MLR). Given that the χ^2^ statistic is sensitive to sample size, alternative fit indices were also considered to assess violations of measurement invariance [39]. Configural (equal factor structure), full metric (equal factor loadings), full scalar (equal factor loadings *and* equal intercepts), and residual error (equal factor loadings, equal intercepts, *and* equal residual error variance) invariance were supported if the configural model showed acceptable model fit and each of the subsequent models showed at least two of: a nonsignificant change in χ^2^ from the previous model, decreases in CFI less than or equal to 0.002 from the previous model, and differences in McDonald’s non-centrality index (NCI) from the previous model below established cut-offs on the basis of the number of items and factors [39]. If a violation of full measurement invariance was detected, modification indices were consulted to examine if partial invariance could be established.

## 3. Results

### 3.1. Frost Multidimensional Perfectionism Scale—Brief

The FMPS-Brief was completed by a total of 708 participants, of whom 299 (42.2%) had a history of self-injury. Of those with a history of self-injury, the most commonly reported methods of self-injury were cutting (*N* = 212, 70.9%), scratching (*N* = 162, 54.2%) and pinching (*N* = 155, 51.8%). 

**Figure 1:**
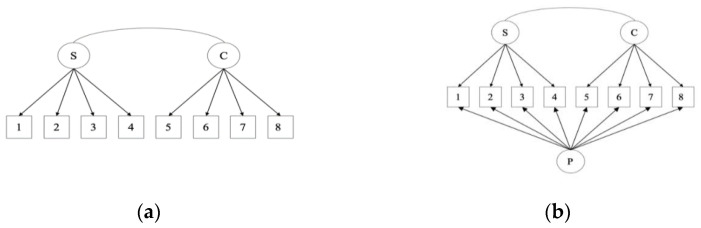
Tested factor structures of the FMPS-Brief; (**a**) Two-factor FMPS-Brief; (**b**) Bifactor FMPS-Brief; note: S = perfectionistic strivings, C = evaluative concerns, P = general perfectionism.

The baseline model fit was assessed for two possible factor structures of the measure: a traditional two-factor structure and a bifactor model (see Figure 1). Each of these models are reported below along with tests of measurement invariance where baseline fit was assessed as adequate.

#### 3.1.1. Two-Factor Solution

The two-factor solution of the FMPS Brief has four items loading onto each subscale for a total of eight items. Baseline model fit was acceptable for the two-factor solution according to CFI and SRMR although the RMSEA was elevated (see Table 2). Configural (M1), metric (M2), scalar (M3), and full residual error (M4) invariance were supported in the two-factor solution according to all considered fit indices (p MLR Δχ^2^, ΔCFI, and ΔNCI; see Table 3). 

Analysis of latent mean differences revealed that individuals with a history self-injury scored higher on both perfectionistic strivings (unstandardized *M*_NSSI_ = 0.76, *Z* = 9.80, *p* < 0.001) and evaluative concerns (unstandardized *M*_NSSI_ = 0.34, *Z* = 4.11, *p* < 0.001) than individuals with no history of NSSI. 

#### 3.1.2. Bifactor Solution

We also assessed baseline model fit for the bifactor model of the FMPS (see Table 2). This model involves a general factor, and two specific factors (perfectionistic strivings and evaluative concerns). The model fit was excellent according to CFI, RMSEA, and SRMR.

Full configural (M1), metric (M2), and scalar (M3) invariance were all supported. Full residual error was not supported (M4), as further analyses revealed higher residual error invariance in item 8 (“I expect higher performance in my daily tasks than most people”) for the group of individuals with a history of NSSI compared to those without a history of NSSI. Allowing this residual error to vary, partial residual error invariance (M4.1) was supported.

There were significant latent mean differences for perfectionistic strivings such that individuals with a history of self-injury scored higher than individuals without a history of NSSI (unstandardized *M*_NSSI_ = 0.67, *Z* = 7.26, *p* < 0.001). Regardless of whether the differential item functioning of item 8 on the general factor was considered (unstandardized *M*_NSSI_ = 0.07, *Z* = 1.27, *p* = 0.203) or ignored (unstandardized *M*_NSSI_ = 0.07, *Z* = 1.19, *p* = 0.234), there were no significant differences in the general factor. There was no significant difference in latent means for perfectionistic concerns between individuals with and without a history of NSSI (unstandardized *M*_NSSI_ = 0.26, *Z* = 1.67, *p* = 0.095). Importantly, if differential item functioning were ignored for item 8 on the perfectionistic concerns factor, it would be erroneously concluded that there were mean differences in perfectionistic concerns (unstandardized *M*_NSSI_ = 0.06, *Z* = 1.98, *p* = 0.047). 

### 3.2. Clinical Perfectionism Questionnaire

The CPQ was completed by a total of 711 participants, of whom 299 (42.1%) had a history of self-injury. Of those with a history of self-injury, the most commonly reported methods of self-injury were cutting (*N* = 212, 70.9%), scratching (*N* = 161, 53.8%) and pinching (*N* = 155, 51.8%).

**Figure 2:**
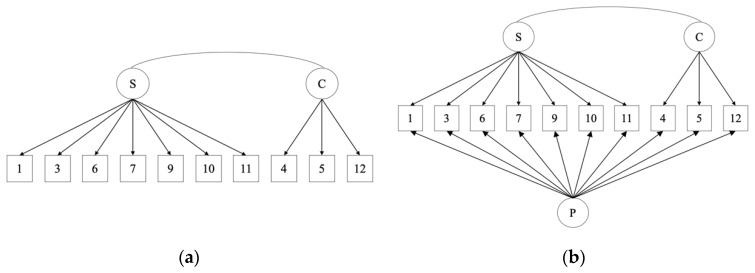
Tested factor structures of the CPQ; (**a**) Two-factor CPQ; (**b**) Bifactor CPQ; Note: S = perfectionistic strivings, C = evaluative concerns, P = general perfectionism.

The baseline model fit was assessed for two possible factor structures of the measure: a traditional two-factor structure and a bifactor model (see Figure 2). Baseline model fit was also assessed for a unidimensional factor as reported by Howell et al., 2020 [17] but was a poor fit to the data (as in Howell et al., 2020) and so no further analysis was conducted using this factor structure. Each of the assessed models are reported below along with tests of measurement invariance where baseline fit was assessed as adequate.

#### 3.2.1. Two-Factor Solution

Using the two-factor solution, the initial model fit was satisfactory according to SRMR, but was unacceptable according to the CFI and RMSEA (see Table 2). Modification indices were reviewed and indicated that allowing item 3 (“Have you been told that your standards are too high?”) and item 10 (“Do you think that other people would have thought of you as a perfectionist?”; Modification index 48.44) to covary would significantly improve model fit. Following these modifications, the baseline model fit was closer to but still not acceptable according to CFI (0.863) and RMSEA (0.099). 

Item 7 had noticeable cross-loadings, and so we tested the model fit without this item. After removing item 7 and allowing items 3 and 10 to covary, model fit was improved such that SRMR was acceptable, although it did not quite reach traditional benchmarks for adequacy according to CFI (0.899) and RMSEA (0.086). Although these model fit statistics were not ideal, we proceeded to evaluate this model for measurement invariance to assess if the measurement was consistent across the groups. 

While configural (M1) and metric (M2) invariance were supported according to two of the three fit indices (ΔNCI and *p* MLR Δχ^2^), scalar (M3) invariance was not supported (See Table 3). Partial scalar invariance was also not foundjk after consulting modification indices.

#### 3.2.2. Bifactor Solution

A bifactor model failed to converge. To explore the source of this failure, the bifactor model was tested for the group with a history of NSSI (*N* = 299) and no history of NSSI (*N* = 412) separately; good fit was observed among participants with no history of NSSI (CFI = 0.930, RMSEA = 0.076 [0.058, 0.094], SRMR = 0.041, χ^2^/df = 3.35). The group with a history of NSSI however would not converge. It is unlikely that this is due to sample size, and we therefore conclude that the bifactor solution is variant across groups. 

## 4. Discussion

Although non-suicidal by definition, NSSI is linked to future suicidal ideation and behaviour and is considered to be an important risk factor for suicide [5]. Perfectionism, associated with suicide risk, also appears to be related to NSSI [16]. However, the nature of cognitive processes which are linked to NSSI may affect how individuals respond to measures of perfectionism. It is critical to test whether current self-report measures of perfectionism are consistent across groups of individuals with and without a history of NSSI. The results of the current study suggest that for the FMPS-Brief, both the two-factor and bifactor structures can measure interpretable between-groups differences in perfectionism. This contributes to growing evidence for the vigour of the FMPS-Brief, with measurement invariance supported across cross-cultural groups [21]. It should be noted that although the bi-factor model demonstrated full residual error invariance, there was differential item functioning which, if ignored, may lead researchers to erroneously conclude that there were significant differences in perfectionistic concerns.

However, findings were considerably less clear regarding the CPQ. This measure had unusually poor baseline model fit even after modification, and comparatively weak internal consistency, which was inconsistent with previous research indicating the measure is psychometrically sound e.g., [25,26]. Importantly, there is no existing research on the measurement invariance of the CPQ across other groups (such as gender or culture). As such, it would be interesting to reproduce the present study in other samples. Nonetheless, measurement invariance was tested on the present data and the pattern of findings indicated that the CPQ was not invariant between groups. Given the poor baseline fit, it is difficult to have confidence in transferability of the CPQ’s psychometric properties outside of the present sample.

The current findings have implications for future research and practice in the context of perfectionism and NSSI. Firstly, the FMPS-Brief appears to be an appropriate tool for use in measuring perfectionism among individuals with a history of self-injury. The decision to use the two factor or bifactor model should be guided by the clinical judgment of the researcher or clinician as well as previous literature [25]. For clinical purposes, a time-effective and psychometrically sound way of measuring perfectionism may be to use the general factor of the FMPS-Brief. Researchers seeking to use the CPQ to make comparisons between individuals with and without a history of NSSI should carefully evaluate the psychometric properties in the samples being assessed. 

There are some limitations which require careful consideration. Given the supporting literature for the CPQ [24,25,26,33,34], these disparate findings suggest these data used in the present study may be anomalous. It is therefore strongly recommended that future research seek to evaluate the measurement invariance of the CPQ with further samples where baseline model fit and reliability are more in line with previously reported estimates. Additionally, we recruited an undergraduate university student sample. This is considered to be appropriate for present study as NSSI is a comparatively common behaviour among university students [40]. However, conclusions about the transferability of these findings to other non-student populations cannot be made. Notably, the sample was predominantly (75.3%) female, consistent with other findings that females are over-represented in research on NSSI. Despite this, evidence about gender differences in rates of NSSI is inconclusive. Although some studies have found that females are more likely to have a history of self-injury than males [41], others have found no significant difference in NSSI prevalence, methods, and severity across genders [42]. This may related to the over-representation of females in samples recruited using undergraduate participation pools. Finally, this research also only evaluated NSSI as a binary outcome (yes or no lifetime history of NSSI). It is worth considering that individuals with a recent history (typically conceptualised as NSSI in the past 12 months) may differ with respect to cognitions and emotional regulation in important ways to individuals with a lifetime history (i.e., has self-injured at least once in their lifetime; [43]). Although we were underpowered to explore this in the present study, future research may build upon these preliminary findings with larger samples to explore invariance of measurement across different frequencies of NSSI.

Given the associations between perfectionism, NSSI, and suicide [5,16], these findings may have implications for measurement in the context of NSSI, but also the literature exploring perfectionism, and suicidal ideation and behaviour. However, further research is required to investigate measurement invariance in these contexts.

## 5. Conclusions

The findings from this study suggest that researchers can use the two-factor model or the general factor from the bi-factor model of the FMPS-Brief to assess differences in perfectionism between individuals with and without a history of self-injury. Where researchers are using the bi-factor model (and not the two-factor model), caution should be exercised when using the evaluative concerns subscale. Given the suitability of the general factor for understanding clinical perfectionism and the measurement invariance of this factor, clinicians seeking to assess perfectionism in populations with lived experience of NSSI are encouraged to use the general factor for simplicity and accuracy. Further research into the measurement of perfectionism using the CPQ is encouraged. Understanding the associations between perfectionism and NSSI is important to guide both our theoretical understanding of the behaviour as well as future prevention and intervention efforts, which may have significant flow on effects in reducing suicide ideation and behaviour. 

## Figures and Tables

**Table 1:** Demographic Information across the Samples.

	FMPS		CPQ	
	*N* = 708		*N* = 711	
	*n*/M	%/SD	*n*/M	%/SD
Female gender	533	75.28%	535	75.20%
Age	20.48	2.21	20.48	2.22
Lifetime history of NSSI	299	42.23%	299	42.10%
Sample means				
Strivings	2.93	1.01	2.41	0.61
Concerns	3.34	1.01	2.39	0.69
General factor	3.13	0.85	2.41	0.56

**Table 2:** Baseline model fit statistics.

	Χ^2^	df	RMSEA [90% CI]	CFI	SRMR	NCI
FMPS (*N* = 708)						
Two factor	140.52	19	0.095 [0.081, 0.110]	0.990	0.025	0.918
Bifactor	48.41	11	0.069 [0.050, 0.090]	0.997	0.016	0.974
CPQ (*N* = 711)						
Two factor	306.14	34	0.106 [0.095, 0.117]	0.837	0.069	0.826
Two factor allowing items 3 and 10 to covary	261.87	33	0.099 [0.088, 0.110)	0.863	0.065	0.876
Two factor (removing item 7 and allowing 3 and 10 to covary)	155.47	25	0.086 [0.073, 0.099]	0.899	0.059	0.894

**Table 3:** Evaluation of measurement invariance in measures of rumination between groups of individuals with and without a history of self-injury.

	Χ^2^	Df	NCI	CFI	Model Comparison	ΔNCI ^a^	ΔCFI ^b^	*p* MLR Δχ^2^
FMPS—two factor								
M1: Configural	159.69	38	0.918	0.959	-	-	-	-
M2: Full metric	162.69	44	0.919	0.960	M1–M2	0.0019 ^+^	0.001 ^+^	0.809 ^+^
M3: Full scalar	167.75	50	0.920	0.961	M2–M3	0.0006 ^+^	0.001 ^+^	0.536 ^+^
M4: Full residual error	181.29	58	0.916	0.959	M3–M4	0.0036 ^+^	0.002 ^+^	0.095 ^+^
FMPS—Bifactor								
M1: Configural	51.32	22	0.980	0.987	-	-	-	-
M2: Full metric	70.94	35	0.975	0.984	M1–M2	0.0046 ^+^	0.003 ^−^	0.105 ^+^
M3: Full scalar	77.73	40	0.974	0.983	M2–M3	0.0012 ^+^	0.001 ^+^	0.237 ^+^
M4: Full residual error	93.96	48	0.968	0.980	M3–M4	0.0057 ^+^	0.003 ^−^	0.039 ^−^
M4: Partial residual error ^c^	80.26	47	0.977	0.985	M3–M4.1	0.0031 ^+^	0.002 ^+^	0.925 ^+^
CPQ								
M1: Configural	172.51	50	0.917	0.900	-	-	-	-
M2: Full metric	176.127	57	0.920	0.903	M1–M2	0.0022 ^+^	0.003 ^−^	0.823 ^+^
M3: Full scalar ^d^	192.93	64	0.923	0.895	M2–M3	0.0063 ^+^	0.008 ^−^	0.019 ^−^

Notes. ^a^ cut-off value for ΔNCI > 0.0069 (FMPS) and ΔNCI > 0.0074 (CPQ) ^b^ cut-off value for ΔCFI > 0.002, ^c^ allowing residual error for item 8 to vary, ^d^ partial scalar was also not supported after consulting modification indices, ^+^ invariance was supported according to the relevant fit statistic, ^−^ invariance was not supported according to the relevant fit statistic.

## Data Availability

The data presented in this study are available on request from the corresponding author.
